# The Rise of Vectored Vaccines: A Legacy of the COVID-19 Global Crisis

**DOI:** 10.3390/vaccines9101101

**Published:** 2021-09-29

**Authors:** Danielle Soares de Oliveira Daian e Silva, Flávio Guimarães da Fonseca

**Affiliations:** 1 Laboratório de Virologia Básica e Aplicada, Departamento de Microbiologia, Instituto de Ciências Biológicas, Universidade Federal de Minas Gerais, Belo Horizonte 31270-901, Brazil; dafonsecaflavio@gmail.com; 2 CT Vacinas, BH-TEC Instituto de Ciências Biológicas, Universidade Federal de Minas Gerais, Belo Horizonte 31310-260, Brazil

**Keywords:** vaccine, SARS-CoV-2, COVID-19, recombinant viral vectors, immunization

## Abstract

The COVID-19 pandemic represents a milestone in vaccine research and development in a global context. A worldwide effort, as never seen before, involved scientists from all over the world in favor of the fast, accurate and precise construction and testing of immunogens against the new coronavirus, SARS-CoV-2. Among all the vaccine strategies put into play for study and validation, those based on recombinant viral vectors gained special attention due to their effectiveness, ease of production and the amplitude of the triggered immune responses. Some of these new vaccines have already been approved for emergency/full use, while others are still in pre- and clinical trials. In this article we will highlight what is behind adeno-associated vectors, such as those presented by the immunogens ChaAdOx1, Sputnik, Convidecia (CanSino, Tianjin, China), and Janssen (Johnson & Johnson, New Jersey, EUA), in addition to other promising platforms such as Vaccinia virus MVA, influenza virus, and measles virus, among others.

## 1. Background

In March of 2020, the World Health Organization (WHO) characterized the public health emergency that had its epicenter in Wuhan (China), later called COVID-19 coronavirus disease 2019 (COVID-19), as a pandemic [1]. The disease, associated with the emergence of severe acute respiratory syndrome coronavirus 2 (SARS-CoV-2), immediately required global efforts by the scientific community in the search for treatments, diagnostic tests and vaccines capable of containing the spread of the virus. In a response time never seen before in the history of medicine, as early as in March of that year, more than 100 vaccine prototypes were in the pipeline [2,3].

Today, more than 10 vaccines are in full use around the globe, including strategies as diverse as their peculiarities in the context of immunization. At the vanguard of vectored vaccines are the recombinant viruses: adaptable, efficient when it comes to antigen heterologous expression, and safe [4]. Viral vector-based vaccines are not new to science, and have achieved considerable success in the veterinary field [5,6,7,8]; however, no viral vector vaccine had ever been licensed for full human use before the COVID-19 pandemic. Nonetheless, the global crisis brought about by the SARS-CoV-2 emergency created the perfect window of opportunity for new technologies to hold their ground and made themselves widespread and useful. Thus, in this review article, we discuss some of the vector vaccines that emerged in the new coronavirus context: their prophylactic potential, their particularities, and the expectations regarding the future of these new technologies.

For this article, a systematic review of available data from clinical trials as well as from published studies contemplating viral vector vaccines for SARS-CoV-2 was performed in order to provide a global analysis of their features and efficacy, especially for the vaccines already approved for use. Search criteria included the words “SARS-CoV-2 vaccine”, “COVID-19 vaccine” and “vaccine vector”.

## 2. The “Trojan Horse” Technology

**Figure 1:**
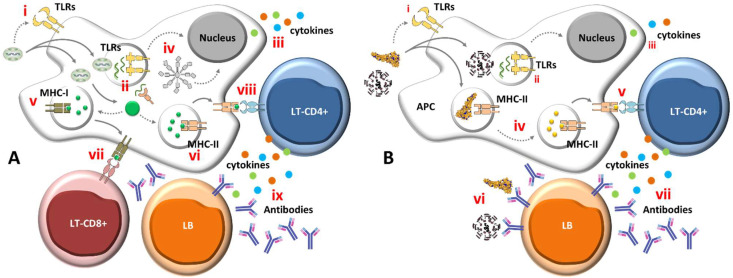
Simplified model of vaccine stimulation of the immune system. (**A**) Immune stimulation by live and/or vectored viral vaccines: after immunization, live vectored immunogens (exemplified here by the modified *Vaccinia virus* Ankara-MVA) enter cells actively (usually through endocytosis-mediated entry), as either somatic and/or antigen-presenting cells (APCs). During its encounter with cells, the virus can activate cell membrane pattern recognition receptors (PRRs) such as Toll-like receptors (TLRs) 2 and 6 [**i**]. Upon entrance, the live vector exposes its nucleic acids and transcribes its genes, including the recombinant transgene (winding green lines); the generated nucleic acids can be sensed by endosomal TLRs (such as TLR3, 7 and 9) or Rig1-Like cytoplasmic receptors (RLRs) [**ii**]. Activation of TLRs and/or RLRs induces the production of pro-inflammatory and antiviral cytokines and chemokines [**iii**]. Infection by the viral vector may induce cell damage, activating NLR-family-pyrin-domain-containing 3 (NLRP3) inflammasome [**iv**], which induces cell apoptosis and cytokine production (mainly if the infected cell is an APC). The transcribed vector-encoded transgene generates the immunogenic protein (large green circle), which can then be proteosome-processed and associated with class I major histocompatibility complex (MHC-I) [**v**] or with class II major histocompatibility complex (MHC-II) in endocytic vesicles [**vi**]. MHC-I molecules loaded with transgenic epitopes translocate to the cell membrane where they are recognized by antigen-specific CD8+ T-cells [**vii**]; the infected cell is killed, liberating antigens in the extra-cellular space. On the other hand, cell membrane-associated, loaded MHC-II molecules are recognized by CD4+ helper T-cells, which secrete cytokines and chemokines and further activate antigen-specific CD8+ T cells and B cells [**viii**]. Stimulated B cells turn into antibody-secreting plasma cells [**ix**] and/or memory B cells. A portion of the stimulated T-cells also become memory cells later on (not shown). Vector-infected cells can also secrete the transgenic protein, which can be picked-up by APCs and induce further immune responses, as depicted in B. Overall, live immunogens are able to equally stimulate both humoral and cell-associated immune responses. (**B**) Immune stimulation by non-live (inert) subunit or inactivated vaccines: upon immunization, antigens and adjuvants present in the formulated vaccine induce cytokine production from local cells, activating and/or attracting APCs to the immunization site. The antigens may further activate APCs after binding to cell membrane TLRs [**i**]. The antigens are phagocyted by APCs and nucleic-acid traces inside the phagosomes may activate endosomal TLRs [**ii**], leading to the production of cytokines and chemokines [**iii**]. The inert antigens are degraded inside the endocytic vesicles, loaded onto MHC-II molecules [**iv**] and presented to CD4+ T-cells [**v**]. Activated CD4+ T-lymphocytes secrete cytokines and chemokines and further activate antigen specific B cells [**vi**], which turn into antibody-secreting plasma cells [**vii**] and/or memory B cells. In general, inert antigens, such as proteins or inactivated viruses, induce potent humoral responses and low to moderate T-cell responses. Activation of CD8+ T-cells by inert antigens occur through alternative pathways that are not depicted in this figure. The stimulation processes depicted in steps i, ii and iii are not as frequent or as potent as stimulation by live immunogens (in **A**), and are depicted in smaller font sizes in (**B**). Receptors and molecules in the diagrams do not necessarily represent their actual molecular structures.

Recombinant viral vectors are generally produced by deleting genes that are essential for the development of a productive and deleterious infection, and the subsequent insertion of genes coding for antigens of interest. Speaking literally, they act as antigen delivery vehicles. Therefore, the golden goal of any viral vector is to achieve some level of a stealth phenotype regarding the host immune response to itself but, at the same time, inducing a strong immune response against the recombinant target antigen. In other words, viral vectors’ basic operating mechanism consists of carrying the target pathogen’s genetic information that is required for the development of a response in its own genome. In this way, the heterologous gene is expressed together with the vector genes during its multiplication cycle, leading to the production of the immunogenic epitopes within the target cell, hence akin to a “Trojan horse” [9,10,11,12] (Figure 1). To accomplish the necessary stealthiness and safety, some of the viral vector’s non-essential genes are deleted and replaced by the desired recombinant genes to be delivered into the host cells [9,13,14,15]. Because many of these non-essential genes have an impact on the vector’s potential virulence, the strategy results in a safe platform, which does not cause illness and that drives into the host cell the genetic information needed to express the recombinant antigen and build a strong immune response. In the COVID-19 context, the main transgene of choice has been the SARS-CoV-2 structural surface glycoprotein-coding gene (spike protein), either whole or partial [16]. Nonetheless, the nucleocapsid protein (N) has also been the target of some studies, either alone or in combination with the S protein [17,18,19].

The vaccine vector technology is not new; however, the COVID-19 crisis has given the viral vectors the opportunity to prove themselves, and three amongst the most widely used anti-SARS-CoV-2 vaccines to date employ adenovirus-based vaccines. Aside from adenovirus vectors, a variety of other viral platforms are currently available, offering versatile genomes adaptable to different insert sizes and the capacity to express, as a rule, any exogenous antigen. The vector choice relies on features such as where, how and when the desired gene will be expressed; the characteristics of the produced recombinant protein; and how it is processed, all focused on the best way to induce a robust response by the vaccinee’s immune system [20]. More importantly, recombinant viral vectors are able to efficiently stimulate both the humoral and cellular branches of the immune response, a necessity to insure the elimination of most pathogens. They act as a natural adjuvant as they undergo the first steps of their multiplication cycle [13], staying active in the host just long enough to trigger immune responses (Figure 1).

Some factors that must be taken into account when choosing a vaccine viral vector are: the viral route of entry; its inflammatory potential; the temporal expression of the transgene; possibility of genome integration; the type of genome; the vector cell/tissue tropism; among other aspects [9,14,21,22]. The promoter driving the expression of the transgene is also a key point when designing a vector, as it will delineate the expression kinetics of the heterologous gene. Constitutive and strong viral promoters are often used, but strong, engineered artificial promoters are also frequently employed [9,23,24,25].

Dosages also need to be carefully evaluated in toxicity and immunogenicity studies before clinical trials, according to the vector. Two-dose vaccines are evaluated according to the lowest dose capable of triggering an immune response. Higher doses are also evaluated, always correlating toxicity, dose volume, route of administration and tolerance [26].

The methods for production and purification of vaccine vectors should also be kept in mind, considering that they need to be scalable to industrial settings [9,27,28,29]. Indeed, most vectors are easily produced and scaled-up [20,30]. The unpredictability of pandemic pathogens requires the scientific community to have at hand tools capable of easily meeting the demand for generation, production and quick licensing [20,31] of vaccines, requirements met by the recombinant vector platforms. Next, we will briefly review the most important COVID-19 vector vaccines that have either been developed or are being developed.

## 3. Some of the Recombinant Viral Vectors Applied to COVID-19

### 3.1. Adenovirus-Based Viral Vectors

#### 3.1.1. Oxford/Astrazeneca Vaccine-ChAdOx1 nCoV-19 (AZD1222 VAXZEVRIA/COVISHIELD)

Adenoviruses are DNA, non-enveloped virus with 30 to 40 kb linear genomes. The E1 and/or E3 gene locus is usually chosen for the recombinant gene insertion, causing deletion of that adenovirus’ gene and ensuring the replication-deficient phenotype [4].

ChaAdOx1, a vector produced from chimpanzee nonreplicating adenovirus-isolate Y25, considered of low seroprevalence in humans, was developed at Oxford University (manufactured by Astrazeneca, Cambridge, United Kingdom) and was already being tested as a Middle East respiratory syndrome (MERS) vaccine when the coronavirus pandemic broke out [32,33,34]. The vector expresses the SARS-CoV-2 structural surface glycoprotein antigen (spike protein) gene and started Phase I clinical trials in the United Kingdom in April of 2020 [35]. From there, the studies were extended to Phase III trials in Brazil and South Africa, involving healthy adults aged 18–55 years, older adults (≥56 years), health-care workers and individuals with comorbidities [35]. The results obtained in four independent clinical trials demonstrated an overall efficacy of 70.4% (after two doses) and 64.1% (after one dose) against symptomatic disease [35]. More recent results, published as non-peer reviewed data at the Astrazeneca media center, indicated that overall efficacy was adjusted to 76% efficacy against symptomatic COVID-19, reaching 85% global efficacy in individuals over 65 years [36]. The new data refer to the NCT04516746 Phase III trial, which enrolled more than 30 thousand participants [37].

Another recent, non-peer reviewed preprint revealed that 180 days after a single-dose vaccination, antibody levels dropped by half in relation to what was seen at the 28-day post-vaccination (antibody titers’ peak). Cellular responses also remained above the determined threshold, indicating a good generation of immunity even with a single dose. A third dose was also evaluated and has been shown to induce a robust boost in the immune responses, even against variants such as Alpha B.1.1.7, Beta B.1.351 and Delta B.1.617.2 [38].

Dosing of ChAdOx1 nCoV-19 vaccine is 5 × 10^10^ viral particles in both applications, plus excipients [35].

#### 3.1.2. SPUTNIK V (Gam-COVID-Vac)

The vaccine developed by Gamaleya National Research Centre for Epidemiology and Microbiology (Moscow, Russia), also known as Gam-COVID-Vac, employs a combination of human nonreplicating adenoviral vectors. These are administrated as a heterologous (i.e., a combination of two different vectors and/or vaccine strategies as part of a same vaccine regimen) prime-boost protocol (21 days interval between doses). The antigen target is the SARS-CoV-2 spike protein expressed by the human adenovirus 26 (Ad26—first dose, 10^11^ viral particles and excipients) and the human adenovirus 5 (Ad5—second dose—10^11^ viral particles and excipients) [39,40]. The use of two adenoviral vectors would potentiate antigen delivery and consequently increase the effectiveness of the immunogen [41]. This is because in a homologous protocol, which uses the same vector in both doses, the antivectorial responses generated upon the first dose could interfere with the vector on the booster dose and reduce the efficiency of the response against the heterologous antigen [42]. So far, Sputnik V is the only vectored vaccine to exploit the use of heterologous prime-boost protocols. Studies employing heterologous regimens composed of mRNA vaccines and adenoviral vectors are underway.

Phase III trials were conducted in Moscow, Russia, employing more than 20,000 adult volunteers over 18 years of age, and showed 91.6% overall efficacy against COVID-19 infection, and 100% against moderate/severe disease [39,40]. According to a recent press release (non-peer reviewed), the analysis of 3.8 million of vaccinated Russians adjusted the overall efficacy of the vaccine to 97.6% [43].

IgG seroconversion after vaccination with Sputnik-V, 21 days after the first dose, was observed in 94% of vaccine recipients. In the presence of pre-existing immunity, the vaccine in the first dose acted as a booster, raising antibody titers higher than for those considered naïve [44], as suggested by the study of Claro and colleagues that mentions that seropositive individuals would not benefit from a second dose of the vaccine [45]. Virus neutralizing activity (VNA) induced by Sputnik was observed in vaccinated people, and it was also effective against some of the variants of concern, with no significant differences against Alpha (B.1.1.7) or Delta (B.1.617.2) [46].

Recently, the Gamaleya Research Institute launched a new version of Sputnik-V: Sputnik Light. The vaccine is a single-dose version using a recombinant Ad26 as a vector. Preliminary results published in the institute media (noon-peer reviewed) revealed an overall efficacy of 79.4%, 28 days after immunization, with induction of IgG neutralizing antibodies and cellular responses [47,48].

#### 3.1.3. Janssen/Johnson & Johnson

The Janssen vaccine, manufactured by Janssen Biotech Inc., a Janssen Pharmaceutical Company of Johnson & Johnson (New Jersey, EUA), is one of the few, to date, available for use as a single shot. The vaccine is based on the nonreplicating human Ad26 expressing the spike protein of SARS-CoV-2, but differently from the other similar vectors (Sputnik and ChAdOx1 nCoV-19), it expresses the antigen in a prefusion-stabilized conformation [49,50]. Such a conformation is probably the ‘ace in the hole’ for this vaccine, since the modification is able to induce higher levels of neutralizing antibodies than the S protein in its native form [50,51,52], which underlays the vaccine’s effectiveness as a single dose. Regarding dosages, the Janssen vaccine is similar to ChaAdOx1 nCoV-19, at 10^10^ viral particles, whereas Sputnik-V employs 10^11^ viral particles [35,39,49].

Phase III trials are being conducted in Argentina, Brazil, Chile, Colombia, Mexico, Peru, South Africa and the United States [49]. The trials have assessed more than 40,000 volunteers and involved healthy adults aged 18–55 years as well as older individuals (60 years or more) [49,53]. The vaccine’s efficacy against moderate COVID-19, 14 days post-vaccination, was calculated to be 66.9%, and 66.1% after 28 days. Taking protection against severe disease as an endpoint, the efficacy was 76.7% at 14 days post-immunization, and 85.4% at 28 days after vaccine administration. After 42 days post-immunization, the adjusted efficacy was 92.4% [49].

Studies on the durability of the immune responses post-vaccination indicated that protective immunity was still observed 8 months after the shot; and the induced neutralizing antibodies were effective against the variants Alpha (D614G, B.1.1.7), Kappa (B.1.617.1), Delta (B.1.617.2), Gamma (P.1), Epsilon (B.1.429), and Beta (B.1.351) [54]. Booster-dose studies have also been announced by Johnson & Johnson, with preliminary results demonstrating an increase in neutralizing antibodies 28 days after the booster in groups aged 18–55 years [55].

#### 3.1.4. Convidecia (Ad-5 nCoV-CANSINO)

The Convidecia vaccine, from CanSino Biologics (Tianjin, China), also based in the human nonreplicating Ad5 and spike protein, was tested in Wuhan, China, in a Phase II clinical trial using a single vaccine dose. A total of 508 adults over 18 years of age were immunized with a single injection dose in different concentrations of viable vectors. The seroconversion was dose-dependent, and its rates were 96% (higher dose) and 97% (lower dose). Interferon production was also observed in a dose-dependent manner in 90% (higher dose) and 88% (lower dose) of the evaluated individuals [56,57]. The results of a Phase III trial (NCT04526990) have not yet been published [58], but the vaccine was approved for use in China.

An overview of the approved adenovirus-based vectorial vaccines against COVID-19 is presented in Table 1.

### 3.2. Poxvirus Viral Vectors-Vaccinia Virus MVA

Modified *Vaccinia virus* Ankara (MVA) was one of the vaccines used in the 1960s and 1970s during the smallpox eradication campaign led by the WHO. This non-replicative *Vaccinia virus* strain is a well-known and successful vector, generated after more than 500 passages of the original Ankara strain in chicken fibroblasts, after which it maintained its immunogenicity, even being unable to productively replicate in mammalian cells [59].

MVA was already on the radar of virologists working on coronavirus vaccines, being tested in combination with the adenovirus ChAdOx1 as a MERS vaccine employing a heterologous prime-boost protocol [60]. Therefore, it was only natural that these two vectors would also emerge as possibilities in the current pandemic context. One of the current strategies is an MVA-SARS-2-S vector expressing full-length S protein, which was able to induce neutralizing antibodies and polyfunctional CD8+ T cells secreting IFN-γ and TNF-α in preclinical studies in mice [61]. This prototype is now undergoing a Phase I clinical trial either as MVA-SARS-2-S [62] or as MVA-SARS-2-ST (expressing a stabilized form of the SARS-CoV-2 spike protein) [63]. When writing this review, only the MVA-SARS-2-S trial was recruiting participants to receive the vaccine in two shots in a 28 day interval; two different immunization doses are being evaluated [62].

García-Arriaza and colleagues also developed two MVA-based vaccine candidates against COVID-19, one named MVA-S, that presented the SARS-CoV-2 spike gene in the MVA’s thymidine kinase (TK) gene locus, and the other named MVA-Δ-S, that had the *Vaccinia virus*-coded immunomodulatory genes *C6L*, *K7R* and A46R deleted in order to increase immune responses [64,65]. Pre-clinical studies in mice, employing either homologous or heterologous prime-boost regimens, showed the triggering of strong cellular and humoral immune responses [64].

Finally, the COH4S1 vaccine candidate is being tested for safety and tolerability in Phase I clinical trials evaluating three different vaccine doses, with the enrollment of 129 participants [66]. This experimental vaccine comprises an MVA vector expressing two major immunodominant antigens, the S and N (nucleocapsid) proteins of SARS-CoV-2. A preclinical study in mice demonstrated a robust induction of neutralizing antibodies against the new coronavirus as well as cellular responses directed at both antigens. The vector was tested in a prime-boost protocol in two mouse lineages (BALB/c and C57BL/6), and the results highlight the importance of both antigens in the context of protective immune responses, with N being a strong and conserved inducer of cell responses, and S being a potent inducer of neutralizing antibodies [17].

#### 3.2.1. Influenza Virus

Influenza virus-based vaccine vectors have the potential to induce cellular and humoral responses both at the mucosal and systemic levels [67,68]. For SARS-CoV-2, it is a clever strategy to associate immunity against the new coronavirus with that of influenza in the same immunization due to the epidemiological and clinical resemblances between coronavirus respiratory diseases and flu. This could even boost vaccine adherence by the overall population. With such an idea in mind, Loes and collaborators designed and constructed an attenuated influenza A virus expressing the receptor binding domain (RBD) of the spike glycoprotein from SARS-CoV-2 [68]. Preclinical studies in mice showed the production of neutralizing antibodies after a single and low dose [68], opening doors to new possibilities of studies in the area.

As this review was being produced, a Phase I clinical trial was recruiting volunteers to test a nasal spray based on an attenuated influenza virus expressing the RBD region of the spike protein, in two doses. The prototype DelNS1-nCoV-RBD LAIV is being tested in two different doses of either 1 × 10^7^ EID_50_ (embryo infectious dose) or 1 × 10^7.7^ EID_50_, in two doses administered 4 weeks apart [69]. The strategy involves a live attenuated influenza virus (LAIV) with the NS1 (non-structural protein 1) region deleted (DelNS1), an important virulence factor and immune evasion determinant [70]. This strategy has been previously tested against H1N1, also intranasally, with a promising immunogenic potential [71].

Intranasal vaccines are promising strategies, as they can induce local immunity in the entry route of respiratory viruses, decreasing virus spread. However, limitations of the strategy are noted: poorly immunogenic antigens that would need adjuvants may not be successful employed by this route; and systemic effectiveness needs to be analyzed and evaluated. Nonetheless, advantages such as the ease of administration are quite powerful arguments that favor the strategy, which is an essential aspect in a mass immunization plan [72].

#### 3.2.2. Measles Virus

Measles virus (MV) is a member of the *Paramyxoviridae* family with a long history in vaccinology, as the current live attenuated measles vaccine has been used safely and efficiently in billions of children worldwide over the last few decades. Therefore, it is no surprise that this live vaccine emerged as a potential vector for the development of anti-SARS-CoV-2 vaccines, despite fears that preexisting immunity to the vector may hamper the approach [73].

As with MVA, MV vectors were also tested as potential vaccine vectors during SARS and MERS emergence, with the coronavirus’ spike glycoprotein being the target in both cases. In a mice preclinical study, the generated anti-SARS MV-based vector induced good levels of neutralizing antibodies and protected the immunized animals from intranasal challenges with the target virus [74]. For MERS, the results were equally promising, with the triggering of cellular and humoral responses and protection of the vaccinated animals upon challenge [75].

These previous studies have paved the way to current attempts at generating a MV-based SARS-CoV-2 vaccine. In the study of Lu and colleagues, several forms of the S protein encoded by the MV vectors were tested in rats and hamsters [76]. The best results were seen when the prefusion S protein was incorporated into the vaccine vector, prompting the induction of anti-SARS-CoV-2 neutralizing antibodies and T cells, and protecting hamsters from SARS-CoV-2 challenge [76].

#### 3.2.3. Vesicular Stomatitis Virus

The vesicular stomatitis virus (VSV) is an RNA virus of the *Rhabdoviridae* family and has been also used as an expression or vaccine vector in the past [77]. Its success story includes the first vaccine approved for human use against Ebola, the ERVEBO (rVSV-ZEBOV-GP, an VSV expressing *Ebola Zaire glycoprotein*), in 2019 [78]. For COVID-19, most of the studies are still in preclinical phases of development.

Yahalom-Ronen and colleagues tested a recombinant VSV-based vector expressing the spike protein of the novel coronavirus in hamsters. Their results demonstrate the induction of neutralizing antibodies after a single-dose, and the previous immunization was able to reduce viral titers in the lungs and nasal shells of challenged animals [77]. This vaccine prototype was undergoing clinical studies in Phases I/II (IIBR-100) at the time of writing of this review, and was being tested in low-, medium- and high-concentration doses employing a prime-boost protocol [79]. The results are yet to be released. A similar strategy was adopted by Case and co-workers, and they reported that a single dose was sufficient to induce the development of neutralizing antibodies in mice and reduce viral load in challenged animals [80].

#### 3.2.4. Lentiviral Vector

An interesting alternative using a non-replicative lentiviral vector (LV) as a SARS-CoV-2 vaccine was proposed by Ku and colleagues, which produced a lentiviral-based vector able to express the SARS-CoV-2 spike protein upon intranasal inoculation. In mouse preclinical studies, immunization with the vaccine prototype induced high levels of neutralizing antibodies after a prime-boost systemic/intranasal combination of doses, and reduced lung inflammation in mice upon challenge with SARS-CoV-2 [81].

A different vaccine candidate based partially on lentiviruses was developed by Shenzhen Geno-Immune Medical Institute (LV-SMENP-DC) and is now in Phases I/II of clinical trials, with the estimated enrollment of about 100 participants. The vaccine is actually composed of dendritic cells (DCs) that have been primed with a lentiviral vector expressing SARS-CoV-2 antigens [82]. This is an approach similar to some strategies used for the development of anti-HIV vaccines [83].

Other vectorial vaccine approaches for SARS-CoV-2 in clinical trials, not mentioned before, are briefly described on Table 2.

## 4. What to Expect from the Immune Responses to Viral Vectors

Basically, there are two types of viral vectors: the replicative and the non-replicative ones. The first ones are able to continue the infection process beyond the first round of cell infection, generating new viable particles that, in turn, will be able to infect new cells, taking the vaccine antigens along with them. The non-replicative vectors, on the other hand, can start their replication cycle, but cannot produce new viral particles, ending their role in the expression of the transgene in those primarily infected cells [90]. One of the advantages of replicative vectors is an infection kinetics similar to that of a natural infection, leading to the potent production of pro-inflammatory mediators [91]. As for the non-replicating vectors, the biggest advantage is undoubtedly their safety profile.

In the host, in the case of replication-deficient vectors, the transgene is expressed for approximately 24 h, the epitopes are presented via the major histocompatibility complex (MHC), innate T and B cells are recruited, and the vector is rapidly eliminated [13]. Additionally, as the immune system is full of pathways and antigen presentation can take place through several of them, we can expect that a good antigen combined with a good vector can summon the right “soldiers” and trigger robust humoral and cellular responses [92].

It remains to be seen whether effective immunity against the novel coronavirus depends fundamentally on antibodies, cellular responses or an effective combination of both, with the last being the most probable answer. Nonetheless, the literature to date suggests that antibodies against the spike protein and its receptor-binding domain are indeed important in eliminating the infection [16,93]. In this scope, the vectors act differently in each component of the immune system, but share the features of inducing a complete and polyfunctional response [20] based on CD4+, CD8+ T cells and antibodies [4].

The heterologous protocol is a proposal that could be better explored with respect to its numerous benefits. For example, the response to adenovirus is serotype-dependent, with Ad5 being more immunogenic than Ad26 and Ad35 [20,94]. Therefore, a combined strategy is quite interesting in order to increase the final recruitment of the host immune components. However, Ad5 is more prevalent in the human population and that may be a problem in regions with high adenovirus prevalence [20,94]. Despite the fact that Ad5 is more immunogenic (potential to trigger an immune response) compared to other adenovirus serotypes, the quality of Ad26 and Ad35 responses seems to be superior, with the induction of long-lived memory CD8+ T cells, better memory cell recall, and greater boost performances. Thus, memory cells induced by Ad26 and Ad35 present a superior survival and polyfunctionality than that induced by Ad5 [94]. As for the MVA vector, although it can be used as a primary vaccine platform, the virus is an excellent memory CD8+ T cell booster [95]. One of the most important success cases involving MVA consists of an Ebola heterologous vaccine regimen based on Ad26 as a prime dose and MVA as a booster. In a clinical trial using this strategy, 99–100% of the participants developed persistent humoral responses which were detectable for up to 1 year, and also robust CD4+/CD8+ responses [96]. This vaccine regimen has been approved for emergency use.

One key downside to the use of vaccine vectors should always be kept in mind: preexisting antivectorial responses. In the case of vector-based anti-SARS-CoV-2 vaccines, for instance, only time will reveal how much the anti-vector immunity against adenoviruses can compromise the further use of the vector, either as future anti-COVID-19 booster shots, or the use of the vector in other vaccine settings [97]. One thing is certain: there is not, nor will there ever be, a perfect vaccine [16].

## 5. Longer Prime-Boost Intervals in Vectored Vaccines

Much has been said about the differences in intervals between the initial dose and the booster shot of vaccines to SARS-CoV-2. In fact, several factors can influence the effectiveness of a booster dose [98]. What really matters is that the booster dose is spaced enough from the first dose in order to avoid interference by the immune response to the initial dose. For AstraZeneca’s vaccine, an eventual delay in the boost shot seems to have been positive [98], as well as for MVA vaccine prototypes [99].

Immunologically, a minimum interval of 2–3 weeks appears to be essential for the development of memory T and B cells with high proliferative capacity, and a delay in the booster dose does not significantly impact the systemic response [98,100]. Indeed, longer intervals favor germinal center reactions. As for T cells, based on the proliferation and differentiation of naïve cells induced after vaccination, an interval of 2 to 3 months between doses is perfect for an optimal response [100]. The same is seen in re-boosting, where a longer interval triggered cellular responses similar to the early vaccine regimens, including the production of cytokines such as IFN-γ, TNF-α and IL-2 [101].

For ChAdOx1 nCoV-19, the protection after the first dose was maintained between 22 to 90 days and better responses were observed after longer intervals between doses (81.3% in a booster ≥ than 12 weeks compared with 55.1% in a booster < than 6 weeks) [102]. Recently, a preprint (not peer-reviewed) reinforced the importance of longer boost intervals by demonstrating that antibody titers after 28 days post-booster dose were higher in groups that were subjected to longer intervals (44 to 45 weeks). Such titers, after 6 months, also remained higher in those groups that received the booster with a longer interval of 15–25 weeks in comparison with the initially proposed standard group that received the dose between 8–12 weeks. [38].

Thus, although the ChAdOx1 nCoV-19 vaccine was the only one to expand the original vaccination booster plan, there may be a trend for the other vectorial vaccines to follow the same path. This will be a natural study approach that will reach both vaccines already under study and others under prospect. The question is and always has been about the response time that pandemic demands.

## 6. Final Remarks

There is a general saying that a good vaccine is a vaccine being used. Viral vectors are certainly at the forefront of the COVID-19 vaccine race, mainly due to the type of responses induced: fast, effective and robust. Fortunately, the COVID-19 crisis created the window of opportunity for human trials employing anti-SARS-CoV-2 vectored vaccines. Before the emergence of COVID-19, viral vectors were authorized for full use only in the veterinary field [5].

However, as with everything new to society, the quick emergency use approval of viral vectors in the COVID-19 context caused estrangement and mistrust. This was further complicated by intense publicization of fake news and misinterpreted data, especially through social media. Indeed, the intense dispersion of misinformation through social media has become a new phenomenon that needs to be curbed now and in future fights against diseases. The trust in adenovirus-based vaccines, for instance, was severely affected by the widespread dispersion of news related to clotting disorders in the vaccinated elderly and in pregnant women [103]. Indeed, reports of patients presenting venous thrombosis and thrombocytopenia after the first shot of the Astrazeneca’s vaccine were published, but the events were, then, proved to be so rare that the benefits of the vaccine still outweigh eventual risks by many orders of magnitude [104,105].

It is also important to eventually compare the efficacy and safety of vectored vaccines with other vaccine strategies used to curb the COVID-19 pandemic, especially the new ones, such as the genetic vaccines (mRNA and DNA vaccines). Pfizer′s mRNA-based immunogen, for example, provides 95% protection after two doses, more effective than any anti-SARS-CoV2 vectored vaccine [106]. Despite the excellent and somewhat surprising performance of the genetic vaccines, with a high efficacy rate and circumventing the problem of anti-vector immunity, we are still in the process of understanding how the vaccines interplay with different components of the immune system, how long protection will last, and many other issues. Therefore, having other new strategies such as the vectored vaccines at hand is certainly prudent. As a clear example, studies have looked into heterologous prime-boost vaccine regimens, using mRNA vaccines as a booster in countries where doses of vectorial vaccines are not available, or the other way around. Indeed, the combination of the different vaccines seems to be a promising strategy.

SARS-CoV-2 is still new to us, and the success of immunizations is closely associated with new discoveries about the infection, the virus’ replication strategies, its antigenic epitopes, and the type of immune responses triggered by it. So far, we are somewhat akin to an escort car driving in parallel to the virus. If it turns to the right, we turn too. It is just over a year into the pandemic, and perhaps only now are the first indications of the immune response’s longevity, either to the infection or to a vaccine, becoming known. Of course, the COVID-19 pandemic brought chaos and has greatly shaken societies and our way of life. Many of these scars, collective or individual, will take years to heal, but there are important and positive legacies as well. The advances toward vaccine development, including the use of vectored vaccines, is one such legacy. Fortunately, this is a path of no return.

## Figures and Tables

**Table 1:** Overview of main results obtained in clinical trials of adenovirus-based COVID-19 vaccines approved for use.

Vaccine	Vector	Target	Trial	Enrollement	Efficacy (Endpoint)	Protocol	References
ChAdOx1 nCoV-19 (AZD1222 Vaxzevria/Covishield)	Replication-deficient simian adenovirus	Full-length structural surface glycoprotein (spike protein-S)	Phase III (NCT04516746)	32,459 participants 18 years to 130 years	76%—symptomatic COVID-19 100%—severe or critical disease and hospitalization 85%—symptomatic COVID-19 in aged 65 years and over	Homologous prime-boost after 28 days	[37,38]
Sputnik V (Gam-COVID-Vac)	Recombinant human adenovirus type 26 (rAd26) and rAd type 5 (rAd5)	Full-length S glycoprotein	Phase III (NCT04530396)	21,977 participants 18 years to 111 years	91.6% 91.8% in older than 60 years 100%—moderate or severe COVID-19	Heterologous prime-boost after 21 days Prime—(rAd26) Boost—(rAd5)	[40,41]
Ad26.COV2.S (Janssen/Johnson & Johnson)	Replication-incompetent human adenovirus 26	Full-length S glycoprotein prefusion-stabilized conformation	Phase III (NCT04505722)	44,325 participants 18 years and older	66.1% after 28 days 85.4%—severe disease after 28 days 92.4% after 42 days	Single dose	[50,54]
Convidecia (Ad5-nCoV) CanSino	Non-replicating human adenovirus type-5 (Ad5)	Full-length S glycoprotein	Phase II (NCT04341389)	508 participants 18 years and older	96–97% dose-dependent	Single dose	[57,58]

**Table 2:** Vectorial vaccine approaches for SARS-CoV-2—other than adenovirus vectors—in clinical trials.

Vaccine	Vector	Target	Trial	Enrollement	Protocol	References
MV-014-212	Respiratory syncytial virus (RSV)	Surface glycoprotein (spike protein—S)	Phase I (NCT04798001)	130 participants	Single-dose or prime-boost intranasal	[84]
Recombinant Newcastle Disease Virus Vectored Vaccine for SARS-CoV-2	Newcastle disease virus	Surface glycoprotein (spike protein—S)	Phase I (NCT04871737)	90 participants	Prime-boost intranasal/intramuscular	[85]
NDV-HXP-S	Newcastle disease virus	Surface glycoprotein (spike protein—S)	Phases I/II (NCT04764422)	460 participants	Prime-boost intramuscular	[86,87]
CVXGA1-001	Parainfluenza Virus Type 5	Surface glycoprotein (spike protein—S)	Phase I (NCT04954287)	80 participants	Single dose intranasal	[88,89]
